# Salmonella enteritis spondylitis of thoracic spine: a case report and review of the literature

**DOI:** 10.1186/s12893-020-00841-5

**Published:** 2020-08-07

**Authors:** Mengmeng Chen, Ruideng Wang, Jianlin Shan, Hai Tang

**Affiliations:** Department of Orthopaedics, Beijing Friendship Hospital, Capital Medical University, No. 95, Yong An Road, XiCheng District, Beijing, 100050 China

**Keywords:** Case report, Salmonella enteritis, Spondylitis, Thoracic spine

## Abstract

**Background:**

Spondylitis is a very common back problem in orthopedics, but is rarely caused by Salmonella enteritidis. We herein reported an uncommon case of thoracic spondylitis caused by Salmonella enteritidis.

**Case presentation:**

A 68-year-old man with high fever was diagnosed as salmonella septicemia initially. His condition was improved after antibacterial treatment. But the symptom of pyrexia was recurred after some days. He was then diagnosed with thoracic spondylitis caused by salmonella enteritidis. After that, he was put on strict antibiotic treatment, and underwent intervertebral lesion debridement, partial rib resection, intervertebral bone fusion and pedicle screw internal fixation. Subsequently, the patient had a significant relief in pain, temperature remained normal, and had no severe complications.

**Conclusions:**

Special attention should be paid to systemic pain and remain cautious to the occurrence of osteomyelitis in patients with Salmonella septicemia. Moreover, the treatment time for using sensitive antibiotics should be sufficient. Surgical treatment should be considered if strict conservative treatment is failed.

## Background

Salmonella spondylitis is a rare disease condition, and it commonly occurs in patients with sickle cell anemia and those in immunocompromised state [1]. We herein presented an immunocompetent case with Salmonella spondylitis who initially developed fever, followed by atypical chest and back pain. This is a rare case of Salmonella enteritis spondylitis of thoracic spine.

## Case presentation

**Fig. 1:**
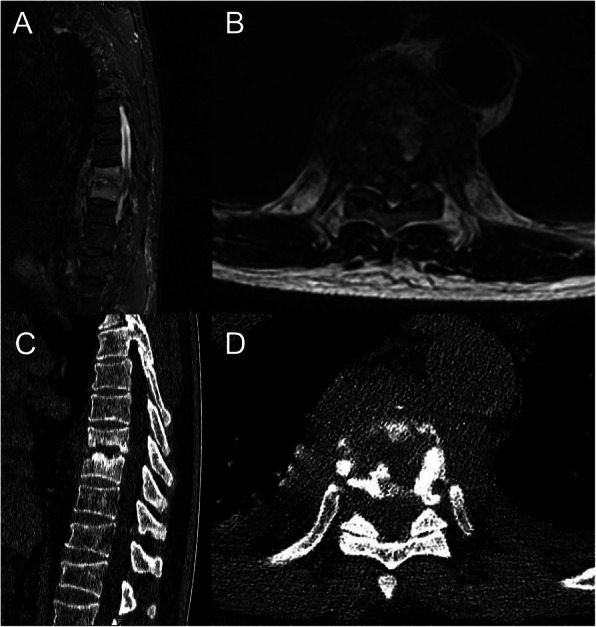
Radiological studies before operation. **a** Sagittal SPIR before operation demonstrated existence of abnormal signals in the intervertebral disk space between T9 and T10; **b** T2 weighted axial image showed well-defined abnormal paraspinal and intraspinal signals; **c** Sagittal CT image showed damaged vertebrae and endplate (T9/T10); **d** CT axial image showed paraspinal and intraspinal soft-tissue mass and partial osteosclerosis

**Fig. 2:**
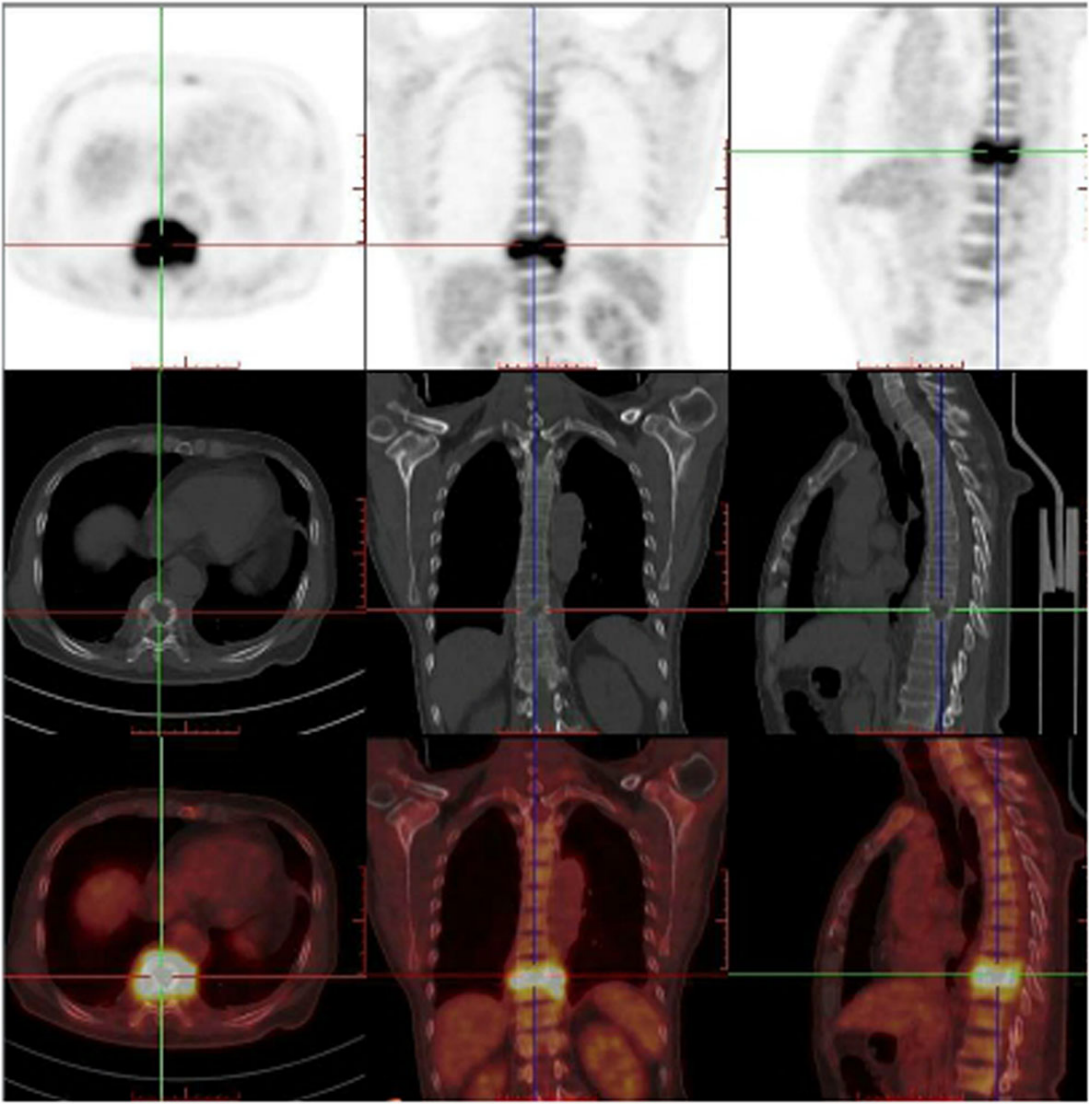
PET-CT images before operation. PET-CT showed changes in T9-T10 vertebrae and intervertebral space, formation of soft tissue masses adjacent to the vertebra, and increased fluorodeoxyglucose (FDG) metabolism

**Fig. 3:**
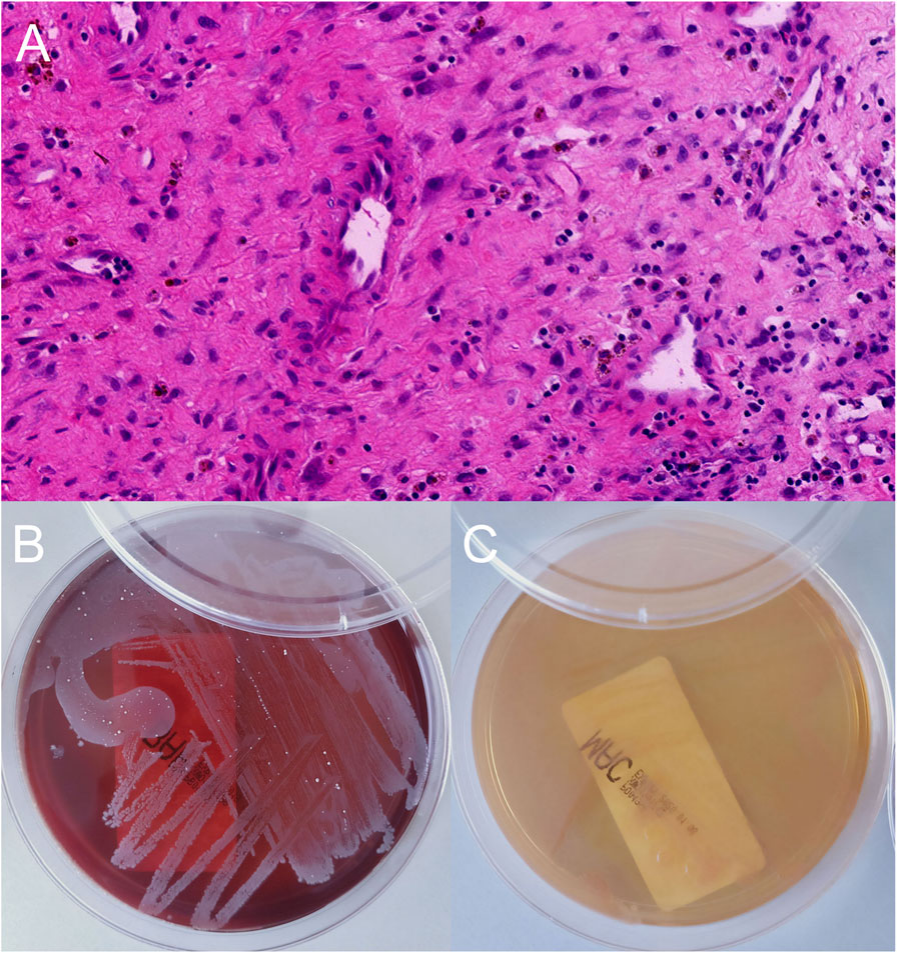
Postoperative histopathology and blood culture. **a** Postoperative histopathology showed inflammatory cell infiltration; **b** In aerobic culture dish, growth of the bacteria was shown; **c** In anaerobic culture dish, no bacterial growth was observed

**Fig. 4:**
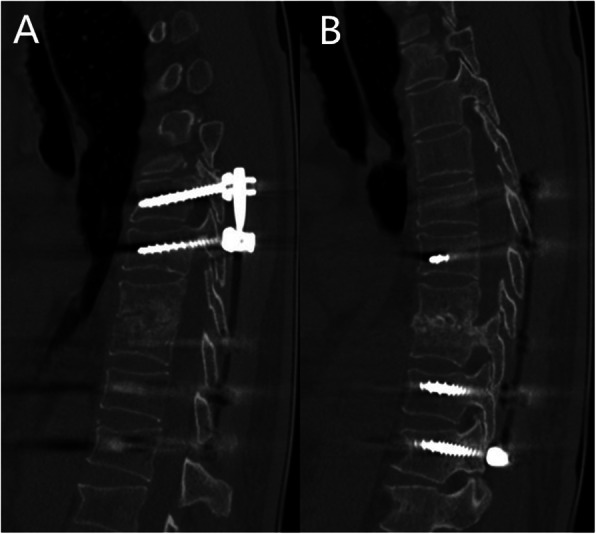
Postoperative CT imaging results after 4 months. **a**, **b** intervertebral fusion in T9-T10 was achieved

A 68-year-old man with high fever and without any apparent cause was prescribed oral moxifloxacin in a community clinic. On day 5, his maximum body temperature reached to 41 °C with shivering, and so he visited the emergency department immediately. On laboratory examination, his white cell count was raised to 11.74*10^9^/L and neutrophil percentage was 92.4%. The results of blood culture indicated Salmonella enteritidis (O9), while Widal test and Weil-Felix test were shown to be negative, which confirmed the diagnosis of Salmonella enteritidis sepsis. The patient was healthy before, and had no history of any other diseases, as well as smoking and drinking history. According to the results of antibiotic susceptibility test, piperacillin/tazobactam was prescribed. His temperature returned to normal after 2 days. He was discharged after maintenance of normal body temperature for 12 days, and was instructed to continue oral cefixime. On day 6 after discharge, he discontinued cefixime by himself and had fever (increased to 38.6 °C) accompanied with chills, fatigue, and night sweats. He also had paroxysmal pain in the left chest and back. He did not visit the hospital and took cefixime at home, and his body temperature was maintained constant at 37–38 °C. However, the patient was admitted after 24 days due to long-term low-grade fever and fatigue. He complained of slight back pain when inquired in detailed. His white cell count was 8.21*10^9^/L and neutrophil percentage was 73.3%. C-reactive protein (CRP) was 45μg/ml, and erythrocyte sedimentation rate (ESR) was 55 mm/h. Blood cultures still indicated Salmonella enteritidis (O9). Spine MRI showed abnormal signal changes at T9-T10 vertebral bone and adjacent soft tissues, and the corresponding spinal cord was compressed. CT scan (Fig. 1) showed infectious lesions. More specifically, bone destruction and sclerotic bone formation were observed, indicating that the course of spondylitis was not short. PET-CT revealed changes in T9-T10 vertebrae and intervertebral space, and formation of soft tissue masses adjacent to the vertebra, increased fluorodeoxyglucose (FDG) metabolism, and spinal tuberculosis was considered (Fig. 2). Anti-mycobacterium tuberculosis antibody and T-spot test were shown to be negative. The final diagnosis confirmed it as thoracic spine infection. Intervertebral lesion debridement, partial rib resection, intervertebral bone fusion and pedicle screw internal fixation were performed. After surgery, the patient experienced a significant decrease in pain. Finally, histopathological results suggested inflammatory cell infiltration and tissue bacterial culture indicated Salmonella enteritidis (O9) (Fig. 3). He underwent treatment with piperacillin/tazobactam for 2 weeks consecutively. His postoperative temperature and white cell count remained normal. Blood cultures were negative twice in a row, so he was discharged from the hospital. Ceftriaxone was prescribed for 3 weeks once a day after discharge. The patient was followed up for 4 months after discharge and showed a good prognosis. From postoperative imaging results after 4 months, intervertebral fusion was achieved in T9-T10 (Fig. 4).

## Discussion and conclusions

Spondylitis caused by Salmonella is rare, which accounted for 0.45% of all osteomyelitis cases [2]. Salmonella osteomyelitis often occurs in immunocompromised patients, and especially those with sickle cell disease [3]. As known, there are several reports on spinal infections caused by Salmonella typhi or Salmonella paratyphi [4, 5]. There are only 10 cases reported with spondylitis of the lumbar (4), cervical (3), and thoracic spine (3) caused by Salmonella enteritidis till date [6, 7]. Also we herein reported a rare case of thoracic spondylitis caused by Salmonella enteritidis (Table 1).

**Table 1:** Thoracic spinal infection cases caused by Salmonella enteritidis

Year	Country	Age/Sex	Presentation	Location	Surgery	Outcome	Pleural effusion
2002	UK	17/Female	Chest and back pain, fever, dysuria, dyspnea,	T9-T11	Only antibiotics	Recovery	Yes
2016	India	47/Male	Back pain, no fever	T7-T8	Debridement, one-stage graft internal fixation	Recovery	No
2016	Greece	57/Male	Back pain, no fever, dyspnea	T11-T12	Debridement, one-stage graft internal fixation	Recovery at first and late onset fatal outcome	Yes
2019	China	68/Male	Back pain, fever	T9-T10	Debridement, one-stage graft internal fixation	Recovery	No

Salmonella is a Gram-negative bacterial intestinal pathogen that spreads through water and food. However, the gastrointestinal symptoms are not presented in some patients [8]. Fever and back pain might be the main symptoms in patients with spondylitis. According to a study, 55% of patients had no symptoms before presenting back pain [5]. Changes in bacterial virulence might explain as to why Salmonella infections occur in immunocompetent people with atypical symptoms [4].

However, Salmonella spondylitis is difficult to distinguish from tuberculosis. The main features of thoracic spinal tuberculosis MRI included well-defined paraspinal abnormal signals, spinal canal and paraspinal abscess, thin and smooth abscess wall [9]. The features of MRI in our case are similar to that of tuberculous spondylitis. As it is difficult to distinguish from tuberculosis, some patients in tuberculosis endemic areas could be misdiagnosed with tuberculosis spondylitis before surgery. However, postoperative pathology revealed infiltration of inflammatory cells and microbiological culture exhibited salmonella infection [10]. Therefore, some measures should be taken to avoid misdiagnosis. Firstly, the blood bacterial culture is critical to identify the pathogens and Widal test might be helpful in that situation. Moreover, neurological symptoms caused by salmonella spondylitis are uncommon, which are different from tuberculosis [11]. In addition, localized puncture is useful for diagnosing it.

Based on antibiotic susceptibility test, a sensitive antibiotic was given through intravenous injection. Although ciprofloxacin, a third generation cephalosporin, ampicillin and chloramphenicol are optional antibiotics that are used as empirical medication, piperacillin/tazobactam was prescribed for treating anti-infection based on antibiotic susceptibility result [6]. For general pyogenic spinal infection, antibiotic treatment for 6–8 weeks is suggested. However, medication time should be prolonged for Salmonella spondylitis, or it might recur [12]. In most Salmonella spondylitis patients, prolonged antibiotics and painkillers cloud result in favorable outcome and complete cure rate reaches 76% [6]. However, there are still 10–20% patients experienced surgical treatment [13].

The surgical indication is still controversial for the Salmonella spondylitis. Most of the literature reported neurologic impairment and spine instability were the surgical indications for Salmonella spondylitis [6, 14]. Besides, Santos added that large intraspinal and paravertebral abscesses and extensive bone destruction were also indications for surgery [12]. In the recent literature, Papaioannou reported a case of thoracic Salmonella spondylitis was performed with corpectomy, mesh cage insertion, and pedicle screw stabilization due to persistent pain, instability and abscess formation [6]. However, in addition to the pathological condition of spondylitis itself, the choice of therapeutic schedule also depended on the lesion location and concomitant diseases. Feng considered that cervical vertebrae was special location which small lesion could easily cause severe pain and neurological deficit, so cervical Salmonella spondylitis was recommended for operative intervention [7]. For thoracic or lumbar lesion, antibiotic treatment was preferred alternative. Chang concluded that Salmonella spondylitis patients with coexisting infection aortic aneurysms exist fatal risk, early surgical intervention could bring about satisfactory prognosis [15].

Although our patient’s temperature continued to be normal for 2 weeks before surgery, the abnormal signals of the spine were still observed on MRI and the CRP/ESR was still higher than the normal, indicating that the infection was not under control after several weeks antibiotic treatment. Postoperative tissue bacterial culture indicated positive for Salmonella enteritidis, and concluded that infection debridement is also essential for this patient.

Two of the four thoracic spondylitis patients had severe pleural effusions and reactive pleuritis as the pleura were in close contact with the thoracic vertebrae. Abnormal signals existed around the thoracic spine, but no pleural effusion occurred in our case. Salmonella septicemia was diagnosed in the early stage, which mainly relied on blood bacterial culture, and so it demonstrated a good treatment effect after strict use of antibiotic therapy. But spinal infection was neglected, which led to deterioration of the disease and spondylitis was diagnosed after 2 months. It was not until after re-admission that the patient noticed back pain when inquired in detailed by the doctor. Therefore, for patients with Salmonella septicemia, sensitive antibiotics should be prescribed and maintain enough medication time. Moreover, attention should be paid to systemic pain and be cautious to the occurrence of osteomyelitis in the early stage. According to a study, PET-CT is able to detect lesions in the early stage before the structure begins to change, but it is an expensive strategy [16].

The following points make our case special: 1. thoracic spondylitis caused by Salmonella enteritidis is very rare; 2. the patient was immunocompetent; 3. the patient had no significant gastrointestinal presentation; 4. missed diagnosis occurred due to atypical manifestations of spondylitis in the early stage.

In conclusion, much attention should be paid to systemic pain and be cautious to the occurrence of osteomyelitis in patients with Salmonella septicemia. If necessary, PET-CT is warranted for early detection of infection sites. Moreover, the treatment time for using sensitive antibiotics should be sufficient. Surgical treatment should be considered if strict conservative treatment is failed.

## Data Availability

All the data supporting our findings are contained within the manuscript.

## Abbreviations

CRP: C-reactive protein
ESR: Erythrocyte sedimentation rate
FDG: Fluorodeoxyglucose

## References

[1] Gupta SK, Pandit A, White DG (2004). Salmonella osteomyelitis of the thoracic spine: an unusual presentation. Postgrad Med J.

[2] D'Souza CR, Hopp PG, Kilam S (1993). Osteomyelitis of the spine due to Salmonella: case report, review of clinical aspects, pathogenesis and treatment. Can J Surg.

[3] Chambers JB, Forsythe DA, Bertrand SL (2000). Retrospective review of osteoarticular infections in a pediatric sickle cell age group. J Pediatr Orthop.

[4] Amritanand R, Venkatesh K, Sundararaj GD (2010). Salmonella spondylodiscitis in the immunocompetent: our experience with eleven patients. Spine (Phila Pa 1976).

[5] Laloum E, Zeller V, Graff W (2005). Salmonella typhi osteitis can mimic tuberculosis. A report of three cases. Joint Bone Spine.

[6] Papaioannou I, Baikousis A, Korovessis P (2017). Multi-foci salmonella enteritis osteomyelitis of thoracic spine with pleural effusion and fatal outcome. A unique case presentation and review of the literature. J Orthop Case Rep.

[7] Feng ZY, Guo F, Chen Z (2014). Literature review and clinical presentation of cervical spondylitis due to salmonella enteritidis in immunocompetent. Asian Spine J.

[8] Govender S, Parbhoo AH, Rasool MN (1999). Salmonella typhi spondylitis. J Pediatr Orthop.

[9] Harada Y, Tokuda O, Matsunaga N (2008). Magnetic resonance imaging characteristics of tuberculous spondylitis vs. Pyogenic spondylitis. Clin Imaging.

[10] Lakshmi K, Santhanam R (2016). Thoracic spinal osteomyelitis due to Salmonella enteritidis in an immunocompetent mimicking tuberculosis. J Neurosci Rural Pract.

[11] Shkurti-Leka K, Kraja D, Leka N (2011). Spondylodiscitis due to Sallmonela in an immunocompetent patient. Med Arh.

[12] Santos EM, Sapico FL (1998). Vertebral osteomyelitis due to salmonellae: report of two cases and review. Clin Infect Dis.

[13] Korovessis P, Repantis T, Iliopoulos P (2008). Beneficial influence of titanium mesh cage on infection healing and spinal reconstruction in hematogenous septic spondylitis: a retrospective analysis of surgical outcome of twenty-five consecutive cases and review of literature. Spine (Phila Pa 1976).

[14] Ikejiri K, Suzuki K, Ito A (2020). Invasive Salmonella Enteritidis infection complicated by bacterial meningitis and vertebral osteomyelitis shortly after influenza a infection in an immunocompetent young adult. J Infect Chemother.

[15] Chang IC (2005). Salmonella spondylodiscitis in patients without sickle cell disease. Clin Orthop Relat Res.

[16] Win Z, O'Flynn E, O'Rourke EJ (2006). F-18 FDG PET in the diagnosis and monitoring of salmonella vertebral osteomyelitis: a comparison with MRI. Clin Nucl Med.

